# Percutaneous closure of a left atrial appendage with relevant suture dehiscence

**DOI:** 10.1007/s12471-016-0933-4

**Published:** 2016-12-19

**Authors:** L. Kleinebrecht, V. Veulemans, A. Polzin, M. Kelm, T. Zeus

**Affiliations:** Division of Cardiology, Pulmonary Diseases and Vascular Medicine, University Hospital Düsseldorf, Düsseldorf, Germany

**Keywords:** Left atrial appendage closure, Suture dehiscence, Percutaneous device

## Abstract

Atrial fibrillation is a widespread disease and highly relevant as it carries an extended risk for ischaemic stroke. Surgical closure of the left atrial appendage is routinely performed during open heart surgery in patients with atrial fibrillation with the aim of thromboembolic protection. In this report we present a successful percutaneous closure of a left atrial appendage, which showed clinically relevant suture dehiscence several years after surgical closure.

Atrial fibrillation is linked to an increased risk of ischaemic stroke. In patients with a CHA_2_DS_2_-VASc score of ≥2, oral anticoagulation is recommended if not contraindicated; in patients with a CHA_2_DS_2_-VASc score of ≥1, oral anticoagulation should be considered according to the current ESC guidelines [1].

The left atrial appendage (LAA) is considered the main source of thromboembolic material. Surgical LAA closure has long been practised during open heart surgery. There is evidence, however, that up to 60% of all surgical closures are unsuccessful while suture and stapler exclusion (i. e. closing the orifice of the LAA cavity) seem to have particularly low success rates compared with surgical excision during which the LAA is actually removed [2]. In addition to that, thrombotic material could be detected in the LAA in up to 40% of all patients who had undergone suture or stapler exclusion [2]. However, there are no controlled clinical data concerning surgical exclusion and only two randomised controlled trials concerning percutaneous closure [3, 4]. Thus, percutaneous device closure may be considered if long-term oral anticoagulation is contraindicated, in accordance with the current ESC guidelines (Class IIb) [1]. Surgical closure of the LAA may also be considered during open heart surgery (Class IIb) [1].

There is evidence that an incomplete surgical and percutaneous exclusion of the LAA is associated with an increased risk for thromboembolic events; [2, 5] however, there is no directive how to treat patients in this constellation.

We present here the case of a 76-year-old male patient, with coronary artery disease, who underwent coronary bypass surgery in 1996. During the following years, he was diagnosed with severe mitral valve regurgitation and atrial fibrillation. Surgical valve repair was performed in 2011. Preoperatively the patient was diagnosed with liver cirrhosis due to chronic hepatitis B infection. With an elevated lifelong bleeding risk due to liver dysfunction, the LAA was surgically closed during valve surgery. The postsurgical course was uneventful and the patient was discharged on aspirin alone. In 2015, the patient suffered an ischaemic stroke. On the stroke unit a thorough search for the cause of the ischaemic event was carried out. Transoesophageal echocardiography (TEE) showed suture dehiscence of the LAA ostium with residual blood flow and smoke within the LAA (Figs. 1 and 2).

**Fig. 1:**
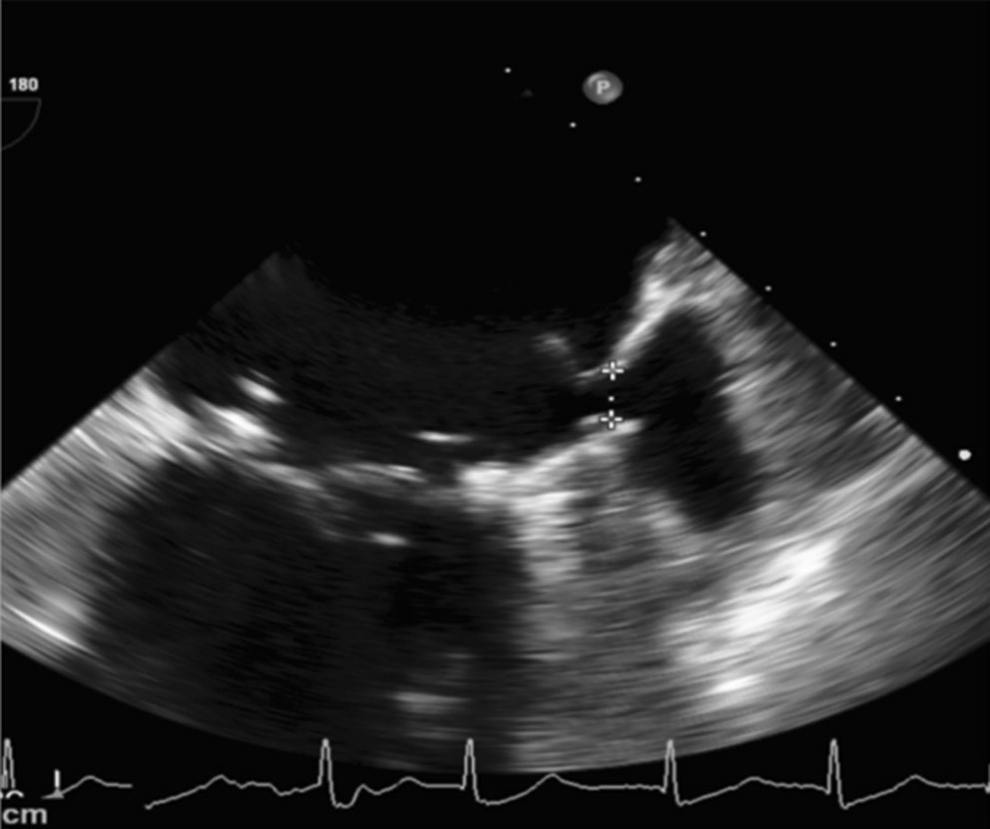
Suture dehiscence (gap 6 × 8 mm)

**Fig. 2:**
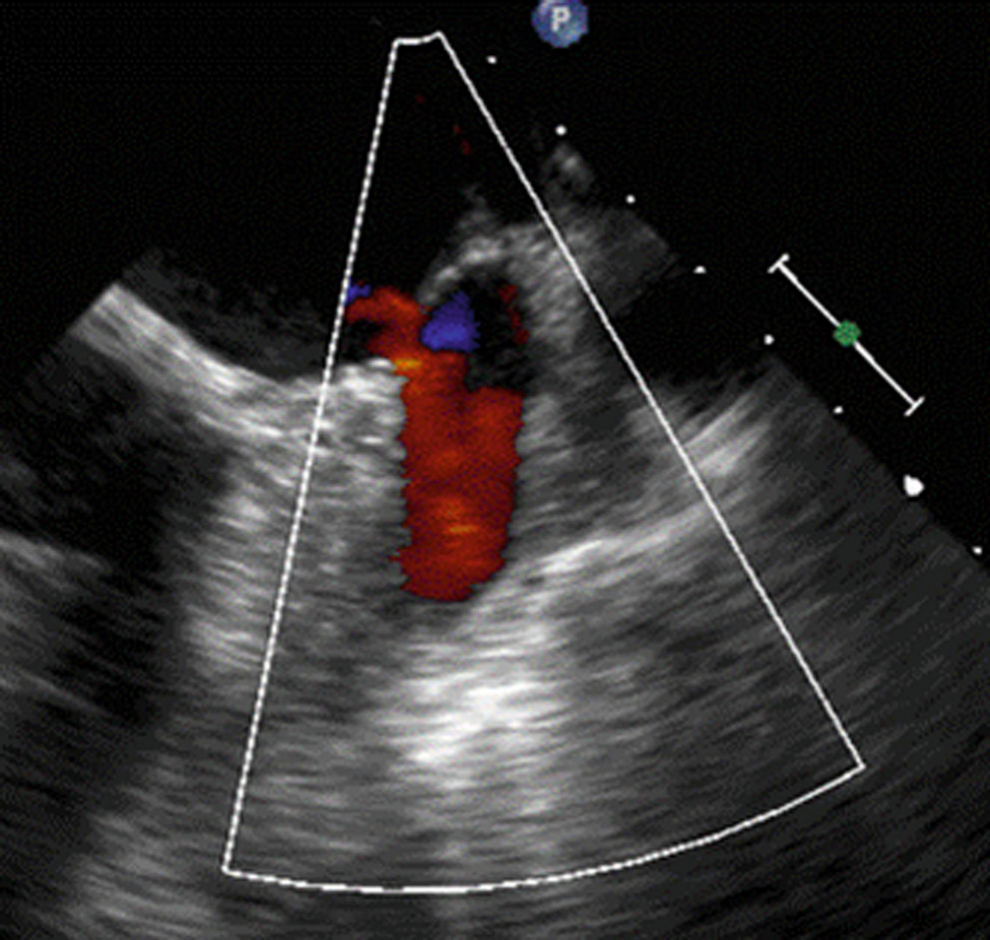
Residual flow in the LAA

The patient had a high risk of a recurrent stroke (CHA_2_DS_2_-VASc score = 6). As life-long anticoagulation was risky (HAS BLED = 5), we decided to perform a percutaneous LAA closure.

The patient agreed after intensive discussion of the procedural risks and alternative therapeutic options. As heparin is applied during the procedure, we waited for six weeks after the stroke to minimise the risk of secondary intracerebral bleeding. The procedure was performed under conscious sedation and with TEE guidance.

The transseptal sheath was inserted through the right femoral vein. With TEE guidance the interatrial septum was punctured in an optimal inferior and posterior position. As the entry of the LAA was too small for the advancement of a pigtail catheter into the LAA, an Amplatz Super Stiff (Boston Scientific) guidewire was positioned in the left upper pulmonary vein. The initial plan was to close the LAA with a 16 mm AMPLATZER Amulet LAA Occluder (St. Jude Medical, SJM). Therefore a 13 F 45°/45° double bend sheath (SJM) was introduced over the stiff wire into the left atrium. Despite good echo guidance, the sheath could not be advanced safely into the LAA. We thought that this was mainly due to the relatively large sheath diameter in correlation to the small, residual LAA ostium and the double bend curve. Therefore we changed to a 9 French standard single bend sheath (45°, SJM). The 9 F sheath could be advanced effortlessly into the LAA. As the oval shaped residual LAA ostium measured 6 × 8 mm, an 8 mm AMPLATZER Septal Occluder (SJM) was chosen. A smaller one was considered to achieve insufficient anchoring. A bigger one was considered to increase the risk of erosion due to the 18 mm distal disc diameter, which would be placed within the LAA. The waist of the 8 mm ASD occluder could be positioned within the LAA ostium and the proximal disc covered the LAA ostium completely. Neighbouring structures were not affected. The occluder was released and TEE showed a good final result (Fig. 3).

**Fig. 3:**
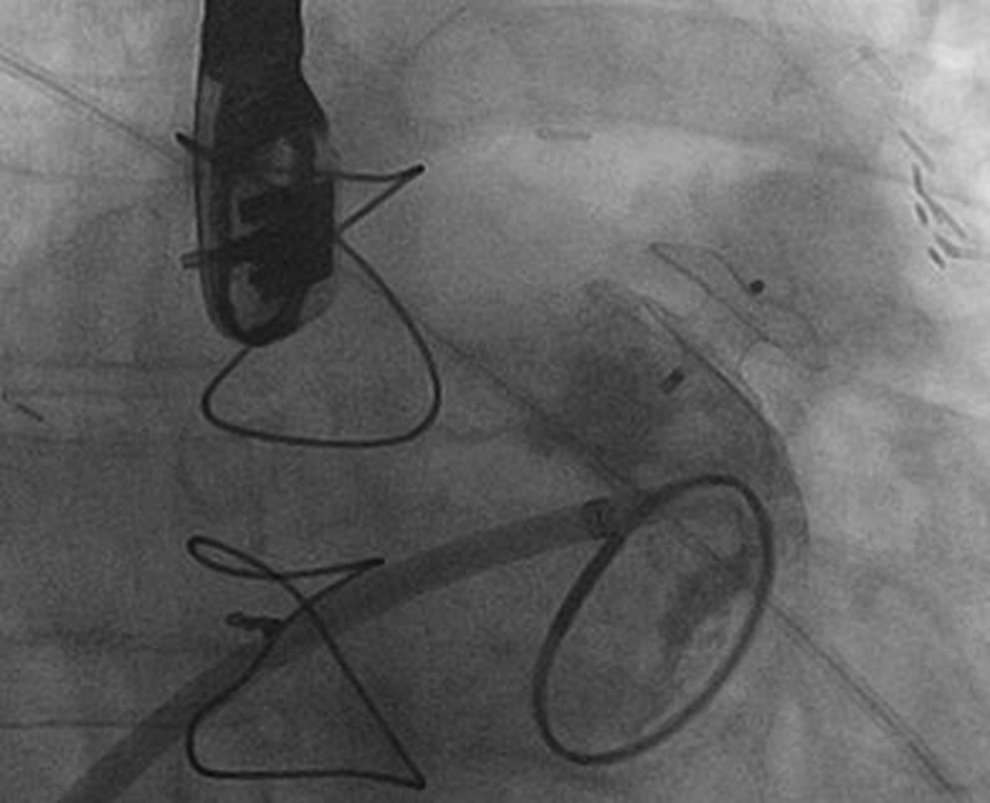
ASD-Occluder placed in the LAA

The patient was transferred to the intermediate care unit, where he spent 24 h free from events. We recommended six months of dual antiplatelet therapy (aspirin and clopidogrel) followed by life-long therapy with aspirin only. Follow-up of 13 months until now has been uneventful (Fig. 4).

**Fig. 4:**
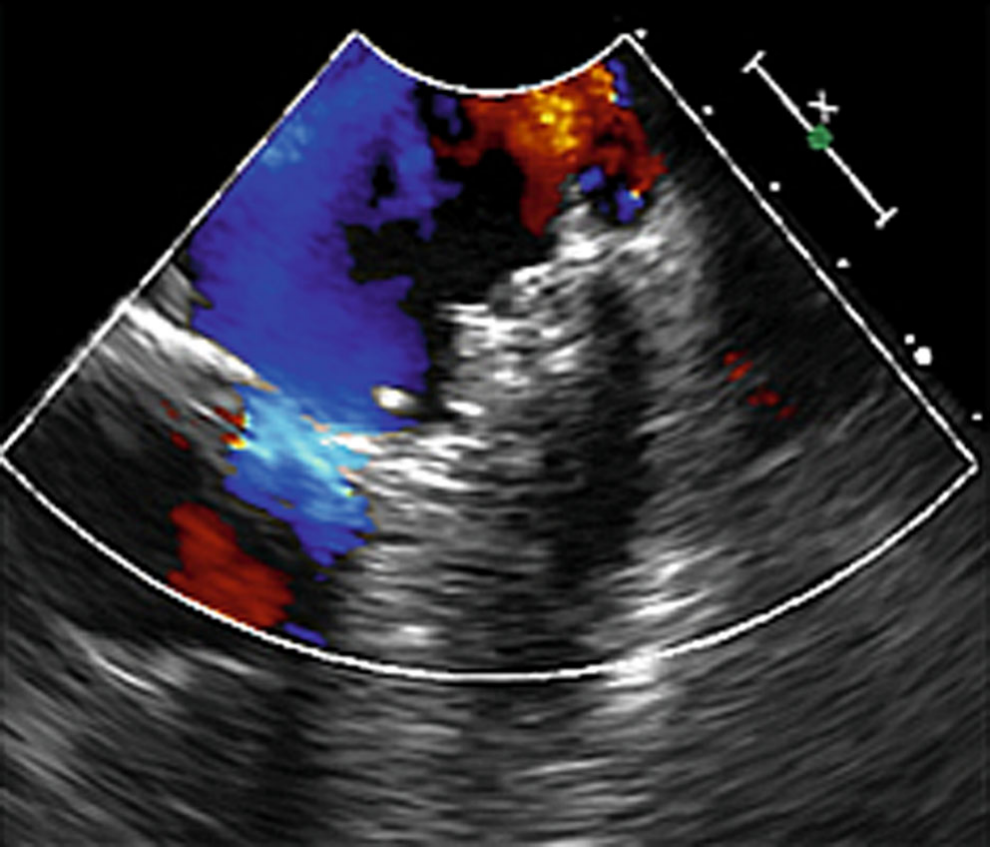
TEE performed three months after the procedure, showing no flow in the LAA

## Summary

Surgical atrial appendage occlusion is often performed during open heart surgery. Insufficient occlusion leads to an elevated risk of thromboembolism. Our patient suffered a stroke several years after a surgically occluded LAA. Transoesophageal echocardiography showed a residual entry to the LAA. Percutaneous closure could be performed with an atrial septal defect (ASD) occluder. Post-interventional imaging showed that the LAA ostium was perfectly sealed.

With regards to the possible impact of ischaemic strokes it seems sensible to perform TEE controls after surgical and percutaneous LAA closure. There is little evidence on when to perform these tests. Concerning percutaneous device closure, TEE is routinely performed three months after the procedure in our clinic. As suture dehiscence in surgically closed LAA might occur later on, it may be necessary to examine these patients even years after the surgery.
